# Aqueous Extract of *Opuntia ficus-indica* (L. Mill) Cladodes Demonstrates Antidiabetic Effect in Normoglycemic and STZ‐Induced Diabetic Rats

**DOI:** 10.1155/bmri/5516794

**Published:** 2026-04-17

**Authors:** Nyaradzo Ellen Masango, Ernest Amponsah Asiamah, Orleans Martey, Justice Kwaku Addo, Samuel Kyei, Alex Boye

**Affiliations:** ^1^ Department of Biomedical Science, School of Allied Health Sciences, College of Health and Allied Sciences, University of Cape Coast, Cape Coast, Ghana, ucc.edu.gh; ^2^ Department of Pharmacology, Centre for Plant Medicine Research, Mampong-Akuapim, Ghana, cpmr.org.gh; ^3^ Department of Chemistry, School of Physical Sciences, College of Agriculture and Natural Sciences, University of Cape Coast, Cape Coast, Ghana, ucc.edu.gh; ^4^ School of Optometry and Vision Science, College of Health and Allied Sciences, University of Cape Coast, Cape Coast, Ghana, ucc.edu.gh; ^5^ Department of Medical Laboratory Science, School of Allied Health Sciences, College of Health and Allied Sciences, University of Cape Coast, Cape Coast, Ghana, ucc.edu.gh

**Keywords:** alpha amylase, alpha glucosidase, diabetes mellitus, hypoglycemia, insulin, *Opuntia ficus-indica*, streptozotocin

## Abstract

**Background:**

*Opuntia ficus-indica* (L. Mill) cladode is traditionally used for food and for the management of diabetes mellitus (DM), but its scientific basis and mechanisms remain underexplored.

**Objective:**

The study assessed antidiabetic effects and mechanisms of action of *O. ficus-indica* aqueous extract (OFIAE).

**Methods:**

Fresh cladodes were blended, centrifuged, and filtered to obtain OFIAE. Phytochemical profiling used colorimetric methods. Antidiabetic potential was evaluated via (1) oral glucose tolerance test (OGTT) in normoglycemic Sprague–Dawley rats pretreated with OFIAE (4, 40, and 400 mg/kg, *p.o.*), metformin, or none; (2) glucose uptake assay in yeast cells with glucose (5, 10, and 25 mM) ± OFIAE (125–1250 *μ*g/mL); and (3) in vitro *α*‐amylase and *α*‐glucosidase inhibition. Experimental diabetes was induced in male Sprague–Dawley rats (STZ–nicotinamide; fasting blood glucose [FBG] > 11 mmol/L). Diabetic rats were treated orally for 28 days with OFIAE (4, 40, and 400 mg/kg), metformin (300 mg/kg), or vehicle. Body weight and FBG were recorded weekly. Glycated hemoglobin (HbA1c) was measured posttreatment. Liver (PAS and H&E) and pancreas (H&E and insulin immunostaining) were histologically examined.

**Results:**

OFIAE contained flavonoids, alkaloids, and phenolic compounds. OFIAE significantly reduced postprandial blood glucose during OGTT compared to control. It enhanced glucose uptake in yeast and inhibited *α*‐amylase (IC_50_ = 411.1 ± 2.23 *μ*g/mL) and *α*‐glucosidase (IC_50_ = 2486 ± 82.0 *μ*g/mL). In diabetic rats, OFIAE reduced FBG and HbA1c levels while increasing body weight compared to the diabetic model. Liver sections showed increased glycogen storage (PAS staining). Pancreatic immunostaining revealed increased insulin expression in islet cells.

**Conclusion:**

OFIAE exhibits antihyperglycemic effects by inhibiting carbohydrate‐digestive enzymes (*α*‐amylase and *α*‐glucosidase), enhancing peripheral glucose uptake, increasing insulin secretion, and promoting hepatic glycogen storage. These findings validate its traditional use and highlight its potential as a complementary therapy for Type 2 DM.

## 1. Introduction

Diabetes mellitus (DM) is a metabolic disorder characterized by hyperglycemia due to insufficient insulin production and/or insulin resistance [1]. According to the International Diabetes Federation (IDF), more than 463 million adults worldwide are living with DM as of 2021, and the records are expected to rise to 700 million people by 2045 [2]. A significant amount of money, amounting to 966 billion USD, was allocated toward diabetes treatment worldwide, posing a global economic burden [3]. The majority of these cases constitute Type 2 diabetes mellitus (T2DM), which is largely connected to insulin resistance, obesity, and sedentary lifestyles [4, 5]. Implicated individuals suffer morbidity and mortality due to DM′s long‐term complications, like cardiovascular diseases, nephropathy, retinopathy, and neuropathy [6]. Conventional pharmacological interventions, such as insulin therapy and oral hypoglycemic agents, play a crucial role in managing diabetes [7, 8]. Nevertheless, several reports have revealed some significant adverse effects associated with these treatments, including hypoglycemia, weight gain, and gastrointestinal disturbances, prompting the need to explore complementary approaches, including herbal medicines [9, 10].

Among the various medicinal plants traditionally used for managing diabetes, *Opuntia ficus-indica* (*OFI*) (L. Mill), well known as prickly pear cactus, has gained scientific attention due to its promising antidiabetic properties [6]. *OFI* (L. Mill) is part of the Cactaceae family and is widely distributed in arid and semiarid regions, particularly in Africa, Latin America, and the Mediterranean [11–13]. Traditionally, diverse parts of the plant, such as the cladodes (pads), fruits, seeds, and flowers, have been used for various therapeutic purposes, including the treatment of diabetes, hyperlipidemia, and inflammation [13]. Phytochemical studies have identified bioactive compounds, such as flavonoids, polyphenols, vitamins, dietary fibers, and alkaloids, which contribute to its hypoglycemic, antioxidant, and anti‐inflammatory properties [14]. Fruit extracts from *OFI* showed a significant reduction in glycemia in Wistar rats and improved glucose uptake by isolated rat hemidiaphragm [15]. A systematic review of the probable hypoglycemic effects of various preparations of the cladodes, boiled, blended, and broiled, showed a similar acute glycemic reduction 120–180 min following cladode intake [16]. Despite the promising findings from preclinical and some clinical studies, further research is required to elucidate the precise molecular mechanisms of *OFI*′s antidiabetic effects in diabetic models.

The present study investigated the antidiabetic properties of *Opuntia ficus-indica* aqueous extract (OFIAE) in vivo (Sprague–Dawley rats) and in vitro, focusing on its impact on blood glucose levels, biochemical parameters, and histopathology of the liver and pancreas, which are key organs in glucose control. Understanding these effects will contribute to the rising body of evidence supporting the use of *OFI* as a natural therapeutic option for diabetes management. This study is particularly significant in the context of the increasing universal burden of diabetes and the need for safer, cost‐effective, and accessible treatment alternatives.

## 2. Materials and Methods

### 2.1. Plant Collection and Preparation

Fresh cladodes of *OFI* (L. Mill) were collected from the Third Ridge area of Cape Coast, Ghana, at geographical coordinates 5.13372° N, 1.24581° W. The plant was authenticated by a herbalist at the Herbarium of the Department of Herbal Medicine, Kwame Nkrumah University of Science and Technology (KNUST), Kumasi, Ghana, and a voucher specimen was deposited under Collection Number KNUST/Hm1/2025/WP002. The cladodes were thoroughly rinsed with distilled water, chopped, and blended into a pulp. The pulp was macerated in distilled water at a proportion of 1:4 (w/v) for 1 h, followed by filtration and centrifugation at 3000 rpm for 5 min. The resulting supernatant, representing a 25% (w/v) aqueous extract, was designated as OFIAE. The extract was freshly prepared weekly and maintained at 4°C until use.

### 2.2. Phytochemical Profiling

#### 2.2.1. Qualitative Phytochemical Screening

The phytochemical examination of OFIAE included the following tests: Alkaloids were tested using Mayer′s test; terpenoids were assessed by Salkowski′s test; tannins were evaluated with the ferric chloride test; phenolic compounds were analyzed via the lead acetate test; sterols were examined using the Liebermann–Burchard test [17]. Additionally, flavonoids and saponins were analyzed following standard methods [18].

#### 2.2.2. Quantitative Phytochemical Analysis

##### 2.2.2.1. Total Flavonoid Content (TFC)

The TFC of the OFIAE was determined using a modified colorimetric method. In summary, 0.5 mL of OFIAE was mixed with 0.3 mL of 5% sodium nitrite solution. Six minutes later, 0.3 mL of 10% aluminum chloride solution was added, and the mixture was kept at room temperature for 6 more minutes, followed by an addition of 2 mL of 1 M sodium hydroxide. The resulting mixture was thoroughly shaken, and the absorbance was determined at 415 nm using a spectrophotometer (T‐9200, United States). A similar preparation without the extract volume was used as a blank, and a standard calibration curve was prepared using 0.01, 0.05, 0.1, 0.2, 0.4, and 0.6 mg/mL of quercetin in deionized water. The TFC was expressed as the milligram equivalents of quercetin per gram of extracts [19].

##### 2.2.2.2. Total Phenolic Content (TPC)

The TPC of OFIAE was determined by mixing 1 mL of OFIAE at increasing concentrations (125, 250, 500, and 1000 *μ*g/mL) with 1.6 mL of deionized water and 0.4 mL of Folin–Ciocalteu reagent. Next, the mixture was incubated for 3 min, followed by the addition of 1 mL of sodium carbonate (20% w/v) and further incubation for 30 min. To obtain the calibration curve, various concentrations of gallic acid solutions (0.10, 0.08, 0.06, 0.04, and 0.02 mg/mL) were prepared as previously described. Absorbances were recorded at 760 nm using a standard spectrophotometer (T‐9200, United States) against a reagent blank. The gallic calibration curve was plotted; absorbance against concentration [20, 21].

#### 2.2.3. LC‐MS Analysis on OFIAE Fractions

Ethyl acetate (20% v/v) was added to freeze‐dried OFIAE, and the two emerging fractions were separately collected. LC‐MS analysis was performed using an Agilent 6460 Triple Quadrupole mass spectrometer with an electrospray ionization. The chromatographic separation was performed on a reverse‐phase column at a temperature of 30°C. Solvents C (water, 80%) and D (acetonitrile, 20%) were used in a gradient elution with a steady flow rate of 0.2 mL/min. The solvent composition was maintained at 30% C and 70% D for the first 10 min, then incrementally adjusted to 80% C and 20% D at Minute 15, which was sustained for 16 min. The autosampler was configured to use wash vial 2 to wash the needle three times with an injection volume of 1.0 *μ*L. The mass spectrometer functioned in positive ionization mode, utilizing a capillary voltage of 4000 V, a nebulizer pressure of 14 psi, a drying gas flow rate of 13 L/min, and a gas temperature of 300°C. Data were obtained in scan mode using a mass range of 70–900 m/z, a scan duration of 0.1 s, and a fragmentation voltage of 70 V. The system was calibrated utilizing the standard ESI tuning file (atunes.tune.xml). Data collection and processing were conducted utilizing Agilent MassHunter software. The liquid chromatograms and mass spectrums were compared with data from the PubChem website and previously published LC‐MS data on *OFI* to identify the possible compounds in OFIAE.

### 2.3. Oral Glucose Tolerance Test (OGTT)

The OGTT was performed as formerly described, with some changes [22]. Briefly, normoglycemic male Sprague–Dawley rats (*N* = 36), fasted overnight, were grouped into six groups: control (administered with distilled water [5 mL/kg, *p.o.*], *N* = 6) model (administered with 6 g/kg D‐glucose and received distilled water 30 min later, *N* = 6), metformin (administered 300 mg/kg, *p.o.*, metformin and 6 g/kg D‐glucose 30 min later, *N* = 6), OFIAE (administered either 4 mg/kg, *p.o.* [*N* = 6], 40 mg/kg, *p.o.* [*N* = 6], or 400 mg/kg OFIAE, *p.o.* [*N* = 6], and 6 g/kg D‐glucose 60 min later). A ball of blood was collected via tail vein pricks and checked for glucose concentration using a standard glucometer (Exactive EQ, Microtec Medical, United Kingdom) at postglucose: 0, 30, 60, 90, and 120 min.

### 2.4. In Vitro Hypoglycemic Analysis

#### 2.4.1. Yeast Cell Glucose Uptake Test

The glucose uptake–enhancing activity of OFIAE was determined as previously described, with minor modifications [21]. A 1% (w/v) suspension of commercial baker′s yeast was prepared in distilled water and incubated overnight at 37°C. Following incubation, the mixture was centrifuged at 4200 rpm for 6 min at 4°C. The pellet was rinsed repeatedly with distilled water and centrifuged until the supernatant appeared clear. The cleaned yeast cells were resuspended in distilled water (1:9 [v/v]).

To assess glucose uptake, 1 mL of the yeast cell suspension was mixed with 1 mL of glucose (5, 10, and 25 mM). The mixture was incubated at 37°C for 1 h, at 4200 rpm for 5 min, and the absorbance of the supernatant was estimated colometrically at 540 nm, which served as a control. Metformin was used as a positive control. The same procedure was repeated with the addition of OFIAE concentrations (125, 250, 500, 1000, and 1250 *μ*g/mL). The mixture was incubated at 37°C for 60 min. The % increase in glucose uptake was computed as follows:

%Glucose uptake=absorbance of control−absorbance of the sample×100 absorbance of control 

where control is the solution containing all reactants except the test sample.

#### 2.4.2. Alpha‐Amylase Inhibitory Activity

The inhibitory activity of OFIAE against alpha amylase was determined photometrically [23]. A substrate solution consisting of 0.5 M Tris‐HCl buffer (pH 6.9), 0.01 M CaCl_2_, and 2 mg/mL of starch (2:1:1) was boiled for 5 min and then incubated at 37°C for 5 min. A reaction mixture was prepared by adding 0.2 mL of OFIAE dissolved in dimethyl sulfoxide (DMSO) (1, 3, 7, 15, 31, 62, 125, 250, 500, and 1000 *μ*g/mL), 1 mL of the prepared starch substrate mixture, and 0.1 mL of porcine pancreatic amylase (0.1 mL in Tris‐HCl buffer) (2 units/mL). The reaction was incubated at 37°C for 10 min, after which 0.5 mL of 50% (v/v) acetic acid was added to stop the reaction. The mixtures were centrifuged at 3000 rpm for 5 min at 4°C, and the supernatant was collected for absorbance measurement. The absorbance of each sample was measured at 595 nm using a spectrophotometer (T‐9200, United States). Acarbose was used as the positive control, and a blank without extract served as the negative control (100% enzyme activity). All experiments were performed in triplicate. The percentage inhibition of *α*‐amylase activity was calculated using the following equation:

%Alpha−amylase inhibitory activity=XA−XB×100XA

where *X*_A_ is the absorbance of the control (100%) enzyme activity and *X*_B_ is the absorbance of the samples.

A graph of percentage inhibition against concentration was plotted and used to extrapolate the IC_50_ of OFIAE and acarbose.

#### 2.4.3. Alpha‐Glucosidase Inhibitory Activity

In vitro *α*‐glucosidase inhibitory activity of OFIAE was performed spectrophotometrically [24]. The substrate solution was prepared by dissolving 15.06 mg of *p*‐nitrophenyl‐*α*‐D‐glucopyranoside (*p*‐NPG) in 10 mL of 0.1 M potassium phosphate buffer (pH 6.8) to obtain a final concentration of 5 mM. An *α*‐glucosidase solution was prepared by dissolving 0.1 unit of the enzyme in 1 mL of potassium phosphate buffer. OFIAE was also dissolved in DMSO at different concentrations: 5, 50, 500, and 5000 *μ*g/mL. The reaction mixture consisted of 20 *μ*L OFIAE, 20 *μ*L *α*‐glucosidase solution, and 40 *μ*L of the substrate. A control reaction was replicated by mixing 20 *μ*L of 0.1 M potassium phosphate buffer (pH = 6.8), 20 *μ*L of *α*‐glucosidase solution, and 40 *μ*L of the substrate. The reaction mixtures were incubated at 37°C for 40 min, after which 80 *μ*L of 0.2 M sodium carbonate in phosphate buffer, pH 6.8, was used to stop the reaction. The absorbance of the reaction mixture was determined at 405 nm using a standard UV spectrophotometer, and the absorbance corresponded to the amount of *p*‐nitrophenol released. The following equation was used to determine the *α*‐glucosidase inhibitory activity:

%Inhibition=Acnt−Aext×100Acnt

where *A*cnt and *A*ext are absorbances of the control and extract at 405 nm, respectively.

A dose–response curve was plotted to perform linear regression analysis and determine the IC_50_ values of the extracts.

### 2.5. In Vivo Antidiabetic Analysis

#### 2.5.1. Animals

Eight‐week‐old male Sprague–Dawley rats (150–230 g) were acquired from the animal house at the Centre for Plant Medicine Research (CPMR) in Mampong‐Akuapim, Ghana. The rats were kept in standard metal cages under ambient conditions, with unrestricted access to commercial feed and water. Ethical consent was granted by the Council for Scientific and Industrial Research (CSIRB) Protocol Number CSIR‐IRB/RPN048/2025.

#### 2.5.2. Diabetes Induction and Treatment

DM was induced in Sprague–Dawley rats using streptozotocin (STZ) (60 mg/kg, *i.p.*) and nicotinamide (110 mg/kg, *i.p.*) [22]. Nicotinamide, dissolved in normal saline and kept on ice, was injected into the rats, followed by STZ dissolved in sodium citrate buffer solution (100 mM, pH 4.5), 15 min later. To prevent death from hypoglycemic shock, the rats were orally administered a 5% glucose solution (2 mL/kg) for 3 days. On the 7th day post‐STZ–nicotinamide injection, the rats were fasted overnight, and blood glucose levels were determined using a standard glucometer (Exactive EQ, Microtec Medical, United Kingdom) after a tail vein prick. Rats with fasting blood glucose (FBG) of at least 250 mg/dL or 11.1 mmol/L were considered hyperglycemic. After induction, the rats were randomly assigned to six groups (*n* = 6) and treated for 28 days (Table 1).

**Table 1:** Animal grouping and treatment.

Group	Treatment
Control	Nondiabetic rats treated with distilled water (5 mL/kg, *p.o.*)
Model	STZ‐induced diabetic rats treated with distilled water (5 mL/kg, *p.o.*)
Metformin	STZ‐induced diabetic rats treated with metformin (300 mg/kg, *p.o.*)
OFIAE (4 mg/kg)	STZ‐induced diabetic rats treated with OFIAE (4 mg/kg, *p.o.*)
OFIAE (40 mg/kg)	STZ‐induced diabetic rats treated with OFIAE (40 mg/kg, *p.o.*)
OFIAE (400 mg/kg)	STZ‐induced diabetic rats treated with OFIAE (400 mg/kg, *p.o.*)

The rats were monitored for weight changes and FBG levels every 7th day. On the 28th day, the rats were sacrificed, and blood samples (5 mL per animal) were collected for analysis. The liver was harvested; the large lobe of the liver was formalin‐fixed for 48 h and processed for periodic acid–Schiff (PAS) test and hematoxylin and eosin (H&E) staining. The pancreas was harvested, formalin‐fixed for 48 h, processed, and stained with H&E and insulin antibody.

#### 2.5.3. Glycated Hemoglobin (HbA1c) Analysis

Blood samples were collected via cardiac puncture and placed in tubes containing 10% ethylenediaminetetraacetic acid (EDTA). An automated blood analyzer (VITROS 5600, Ortho Clinical Diagnostics, Mexico) was subsequently used to determine HbA1c (estimated as a percentage).

#### 2.5.4. Liver Function Analysis

Five milliliters of the blood samples was collected in plain tubes and allowed to clot for 7 min. To obtain serum (2 mL), the clotted blood samples were centrifuged at 3000 rpm for 10 min. Liver function tests were conducted using a semiautomated clinical chemistry analyzer (VITROS 5600, Ortho Clinical Diagnostics, Mexico) at the Cape Coast Teaching Hospital, Ghana. The tests included alanine aminotransferase (ALT), alkaline phosphatase (ALP), protein levels, and bilirubin levels.

#### 2.5.5. Histological Examination

##### 2.5.5.1. H&E Staining

The largest lobe of the liver and pancreas was harvested, rinsed in normal saline, and instantly fixed in 10% neutral buffered formalin for at least 48 h. The fixed tissues were dehydrated through a graded series of ethanol, cleared in xylene, and embedded in paraffin wax. Sections of 5 *μ*m thickness were cut with a microtome and picked onto glass slides. The slides were deparaffinized, rehydrated, and stained with hematoxylin for nuclear visualization and eosin for cytoplasmic and extracellular matrix contrast. After staining, the sections were dehydrated, cleared, and cover‐slipped using DPX Mountant. Four stained slides from each group were observed under a light microscope for histopathological variations. Photomicrographs were taken using an Olympus microscope (Model CXC41) with an attached camera (AmScope MD500).

##### 2.5.5.2. PAS Staining of the Liver and Glycogen Estimation

The paraffin sections of the liver (5 *μ*m thick) were deparaffinized, rehydrated, and treated with 0.5% periodic acid solution for 5 min. Afterwards, the slides were washed in distilled water and stained with Schiff′s reagent for 15 min in the dark. After thorough washing under running tap water to develop the characteristic magenta color, the sections were counterstained with hematoxylin, dehydrated, cleared in xylene, and mounted with DPX. For each animal, five photomicrographs of the liver devoid of portal triad and central vein were taken at ×400 magnification using an Olympus microscope (Model CXC41) with an attached camera (AmScope MD500). Quantitative analysis of hepatic glycogen deposition was performed using ImageJ/Fiji software (National Institutes of Health, United States). Each photomicrograph was uploaded into the ImageJ/Fiji console. A 4 × 4 rectangle of dimensions was generated using a grid of area per point 2800 square pixels and then used to generate five nonoverlapping sections. Each sectioned image was deconvoluted using the default PAS vectors, the red component was inverted, and automatic thresholding was applied to identify PAS‐positive regions. The PAS‐positive area was expressed as a percentage of the total tissue area (%). Mean values per animal were calculated and used for statistical analysis.

##### 2.5.5.3. Immunohistochemical Staining of the Pancreas

Paraffin‐embedded pancreatic tissue sections were stained using an insulin monoclonal rabbit anti‐insulin antibody and goat antirabbit as a secondary antibody (EnoBiotech Ltd, China; 1:200 dilution). Antibody preparation and staining procedures adhered to standard immunohistochemical protocols, with primary antibody incubation carried out overnight at 4°C [25]. Photomicrographs of four islets of Langerhans from each animal were taken at ×400 magnification using an Olympus microscope (Model CXC41) with an attached camera (AmScope MD500). For each animal, six nonoverlapping fields containing pancreatic islets were analyzed. Threshold values of insulin‐positive areas were determined using Fiji/ImageJ software and expressed as the average area of immunoreactive cells (square micrometers) per square millimeter of islet tissue. A uniform thresholding protocol was applied to all images to ensure consistency during islet segmentation.

### 2.6. Statistical Analysis

Data were examined using GraphPad Prism software (Version 9.0). Results are shown as mean ± standard error of the mean (SEM). Statistical significance was assessed using one‐way analysis of variance (ANOVA), followed by Dunnett′s post hoc multiple comparison test. Differences were considered statistically significant at *p* < 0.05 (95% confidence interval). Significant difference between the control group and the model group is shown by ^+^ *p* < 0.05 (control vs. model). Significant difference between the model group and the treatment group is shown by  ^∗^ *p* < 0.05 (model vs. treatment groups).

## 3. Results

### 3.1. Phytochemical Profiling and In Vitro Antioxidant Capacity of OFIAE

**Figure 1:**
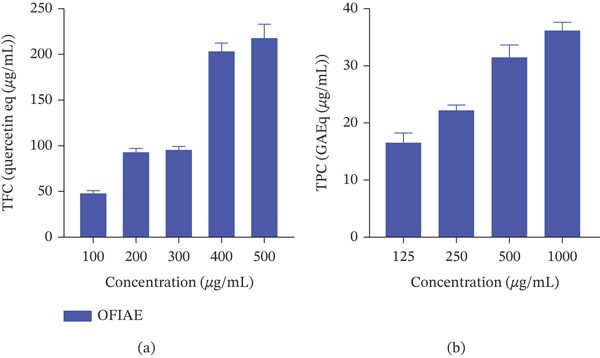
Quantitative analysis of total flavonoid content (TFC) and total phenolic content (TPC) of OFIAE expressed as (a) quercetin and (b) gallic equivalences, respectively.

Qualitative phytochemical screening of OFIAE revealed the presence of flavonoids, saponins, tannins, alkaloids, terpenoids, sterols, and phenolic compounds, all known for their pharmacological activities. Quantitative TFC and TPC increased in a concentration‐dependent manner concerning their quercetin and gallic acid equivalences, respectively (Figure 1).

### 3.2. LC‐MS Profiling of OFIAE

**Figure 2:**
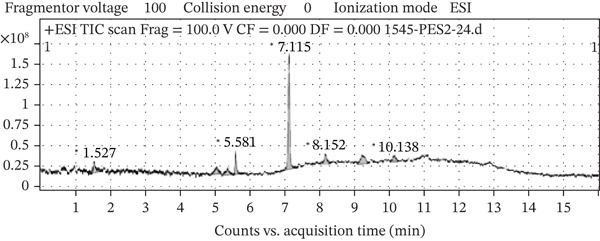
LC‐MS chromatogram of the ethyl acetate fraction of OFIAE. The representative total ion chromatogram (TIC) shows peaks corresponding to eight detected phytocompounds.

**Figure 3:**
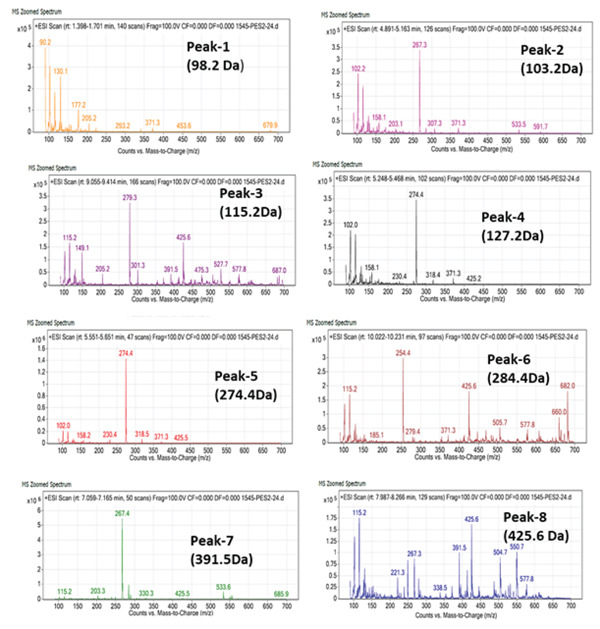
Mass spectra of eight identified phytocompounds in the ethyl acetate fraction of OFIAE.

**Figure 4:**
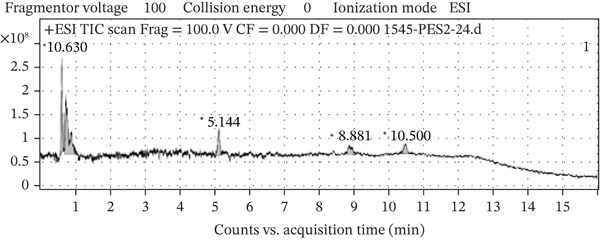
LC‐MS chromatogram of the aqueous fraction of OFIAE. Representative total ion chromatogram (TIC) showing five major peaks.

**Figure 5:**
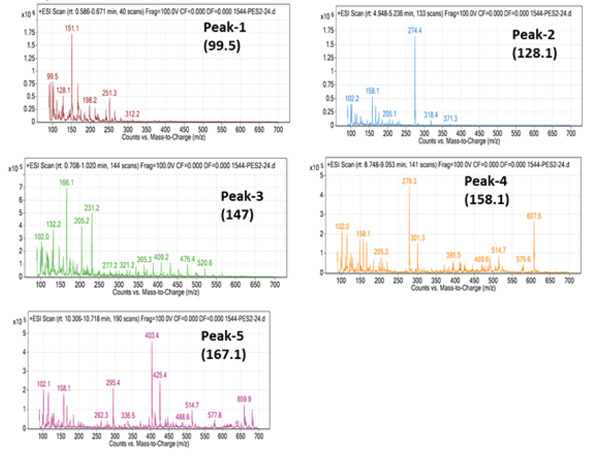
Mass spectra of the five phytocompounds identified in the aqueous fraction of OFIAE.

The two fractions from OFIAE revealed a total of 13 compounds. The data were compared to existing literature to predict some of the phytochemical constituents in OFIAE (Table 2). The LC‐MS analysis of the ethyl acetate fraction yielded eight major peaks corresponding to distinct compounds according to their retention times and mass‐to‐charge (*m*/*z*) ratios. Tentatively identified compounds included phenolic acids (caffeic acid and vanillic acid), flavonoids (kaempferol, quercetin, and isorhamnetin), and sterols (*β*‐sitosterol, linoleic acid, and palmitic acid) (Figures 2 and 3). The aqueous fraction showed five prominent peaks with retention times varying from 0.63 to 10.5 min (Figure 4). The corresponding *m*/*z* values suggest the presence of additional phenolic or flavonoid‐type compounds (Figure 5). Tentatively identified compounds included some similar compounds identified in the ethyl acetate fraction, such as gallic acid, quercetin, and isorhamnetin (Table 2). Other compounds were also tentatively identified in the aqueous fraction, including phenolic acids (piscidic acid and ferulic acid), flavonoids (rutin and nicotiflorin), and fatty acids (oleic acid and campesterol).

**Table 2:** Summary of retention times, exact *m*/*z* values, and suggested phytocompounds identified in OFIAE.

Compound	RT (min)	Major m/z values (from MS)	Possible compound class	Suggested candidates	Notable activities
*Ethyl acetate fraction*					
Peak‐1	1.527	90.2, 91.1, 98.2, 102	Small phenolic/acid	Caffeic acid [26]	Antioxidant, hypoglycemic
Peak‐2	5.041	103.2, 115.2, 117.1, 130.1	Phenolic acid/flavonoid	Vanillic acid derivative [27]	Anti‐inflammatory
Peak‐3	5.366	115.1, 127.2, 130.2, 158.1	Flavonoid glycoside	Isorhamnetin [28]	Antidiabetic, antioxidant
Peak‐4	5.581	115.1, 127.2, 130.2, 158.1	Flavonoid glycoside	Kaempferol‐3‐*O*‐glucoside [29]	Insulin sensitizer
Peak‐5	7.115	115.2, 130.1, 230.4, 274.4	Flavonoid or betanin	Quercetin, indicaxanthin [13]	Antidiabetic, antioxidant
Peak‐6	8.152	267.4, 268.3, 284.4, 285.3	Fatty acid/sterol	Linoleic acid [30]	Anti‐inflammatory
Peak‐7	9.209	267.3, 391.5, 425.6	Sterol or triterpene	*Β*‐sitosterol, campesterol [30]	Antioxidant, antidiabetic
Peak‐8	10.138	279.3, 301.3, 425.6	Sterol	Palmitic acid [31]	Antimicrobial
*Aqueous fraction*					
Peak‐1	0.63	90.6, 94.2, 99.5, 102.1	Phenolic acid	Gallic acid, piscidic acid [32]	Anti‐inflammatory, antidiabetic, antioxidant
Peak‐2	0.756	111, 128.1, 151.1, 167	Phenolic acid/flavonoid	Ferulic acid, isorhamnetin glycoside [32]	Neuroprotective, antidiabetic
Peak‐3	5.144	130.1, 132.2, 147, 116.1	Flavonoid	Quercetin, rutin, nicotiflorin [33]	Antidiabetic, cardioprotective, hepatoprotective
Peak‐4	8.881	115.2, 158.1, 167.1, 176.1	Flavonoid/sterol	Isorhamnetin, kaempferol, *β*‐sitosterol [34]	Antioxidant, antidiabetic, immunomodulatory
Peak‐5	10.5	158.1, 167.1, 279.3, 301.3	Fatty acid/sterol	Linoleic acid, oleic acid, campesterol [35]	Anti‐inflammatory, antioxidant, cholesterol‐lowering

### 3.3. Effects of OFIAE on Glucose Tolerance (OGTT) in Normoglycemic Rats

**Figure 6:**
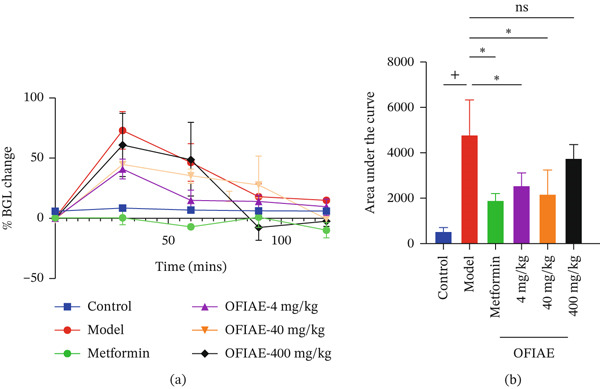
Effect of OFIAE on oral glucose tolerance test (OGTT) in normoglycemic rats. (a) Rats were administered OFIAE (4, 40, or 400 mg/kg), metformin (300 mg/kg), or 5 mL/kg distilled water (control), 30 min before glucose challenge (6 g/kg). Blood glucose concentration (BGC) was measured at 0‐, 30‐, 60‐, 90‐, and 120‐min postglucose challenge (6 g/kg, *p.o.*). (b) The corresponding AUC of glucose clearance under the various treatments. A one‐way ANOVA with Dunnett′s post hoc test was used to compare groups. ^+^ *p* < 0.05 (control vs. model);  ^∗^ *p* < 0.05 (model vs. treatment groups). Each point is the mean ± SEM, *N* = 6.

The capacity of OFIAE to decrease postprandial glucose levels was assessed in normoglycemic rats using the OGTT. Figure 6a illustrates the effect of OFIAE on glucose tolerance in normoglycemic rats following oral glucose loading. Baseline blood glucose levels (0 min) were comparable across all groups before glucose administration (*p* > 0.999). Oral glucose loading produced a rapid increase in blood glucose levels in the control group, with peak glucose concentrations observed at 30 min, followed by a gradual decline over the 120‐min period. Pretreatment with OFIAE (4, 40, and 400 mg/kg) attenuated the postprandial rise in blood glucose and enhanced glucose clearance over time when compared with the model group (*p* < 0.05). Notably, OFIAE‐treated rats exhibited a faster reduction in blood glucose levels from 60 to 120 min postglucose loading, indicating improved glucose tolerance. The reference drug, metformin (300 mg/kg), produced a more pronounced reduction (*p* < 0.05) in blood glucose levels throughout the time course compared to the model group.

To quantify the overall glycemic response during the OGTT, the area under the glucose–time curve (AUC) was calculated (Figure 6b). OFIAE treatment resulted in a significant reduction in AUC compared with the model group (*p* < 0.05), confirming an overall improvement in glucose handling. The reduction in AUC was comparable, though less pronounced, to that observed with metformin. The improved glucose clearance observed following OFIAE administration suggests enhanced peripheral glucose utilization or delayed intestinal glucose absorption, indicating a potential role of the extract in regulating postprandial glycemia.

### 3.4. Yeast Cell Glucose Uptake Assay

**Figure 7:**
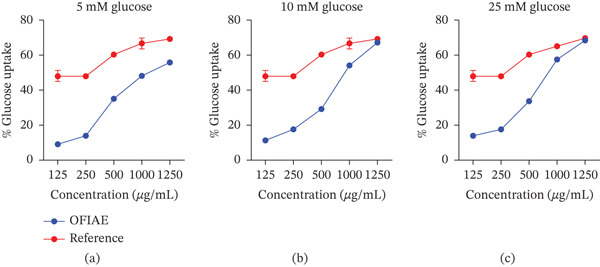
Effect of OFIAE on glucose uptake in yeast cells. Yeast cells were incubated with varying concentrations of OFIAE (125–1250 *μ*g/mL) at varying glucose concentrations. Glucose uptake was quantified spectrophotometrically. Each plotted point is the mean ± SEM, *n* = 3 replicates: (a) 5 mM glucose, (b) 10 mM glucose, and (c) 25 mM glucose.

The control (yeast cells and glucose only) showed an absorbance of 0.94, and this was significantly reduced in the OFIAE‐treated groups (*p* < 0.05) at all glucose concentrations. OFIAE markedly increased glucose absorption in a concentration‐dependent manner, at all tested glucose strengths: 5, 10, and 25 mM (Figure 7). The maximum glucose uptake was observed at OFIAE 1250 *μ*g/mL (70.2%), comparable to that of metformin, the reference drug. Enhanced glucose uptake in yeast cells suggests that OFIAE may facilitate cellular glucose utilization, supporting its potential role in improving peripheral glucose handling.

### 3.5. Inhibition of Digestive Enzymes

**Figure 8:**
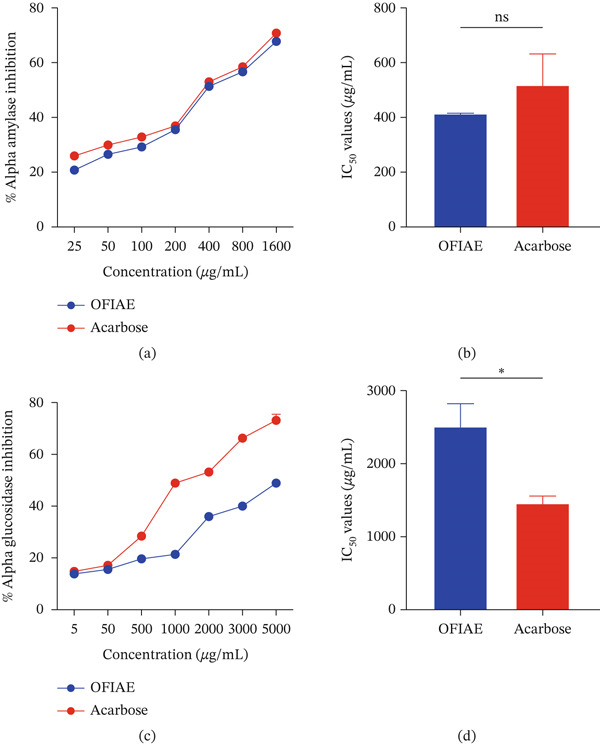
Effect of OFIAE on *α*‐amylase and *α*‐glucosidase activity. (a) Percentage (%) inhibition of *α*‐amylase incubated with OFIAE (25–1600 *μ*g/mL). (b) Corresponding IC_50_ values of OFIAE and acarbose for *α*‐amylase inhibition (411.1 ± 2.23 *μ*g/mL). (c) Percentage (%) inhibition of *α*‐glucosidase incubated with OFIAE (5–5000 *μ*g/mL). (d) Corresponding IC_50_ values of OFIAE and acarbose for *α*‐glucosidase inhibition (2486 ± 82.0 *μ*g/mL).

OFIAE inhibited *α*‐amylase and *α*‐glucosidase, exhibiting maximum inhibition values of 70% and 48.5%, respectively (Figure 8a,c). The corresponding IC_50_ values for *α*‐amylase were 411.1 ± 2.23 *μ*g/mL (OFIAE) and 516.3 ± 6.61 *μ*g/mL (acarbose) (Figure 8b). The corresponding IC_50_ values (Figure 8d) for *α*‐glucosidase were 2486 ± 82.0 *μ*g/mL (OFIAE) and 1446 ± 59.3 *μ*g/mL (acarbose). No statistically significant difference between OFIAE and acarbose was observed for *α*‐amylase inhibition (*p* = 0.189), whereas a statistically significant difference was observed for *α*‐glucosidase inhibition (*p* = 0.006), indicating a stronger inhibitory effect of OFIAE on *α*‐amylase. The inhibitory activity of OFIAE against *α*‐amylase and *α*‐glucosidase was moderate when compared with the standard inhibitors used in this study. However, the observed effects were concentration‐dependent and consistent with the expected activity profile of crude plant extracts. This indicates that the OFIAE may delay carbohydrate digestion and reduce postprandial glucose spikes.

### 3.6. Effects of OFIAE on Weekly FBG in Diabetic Rats

**Figure 9:**
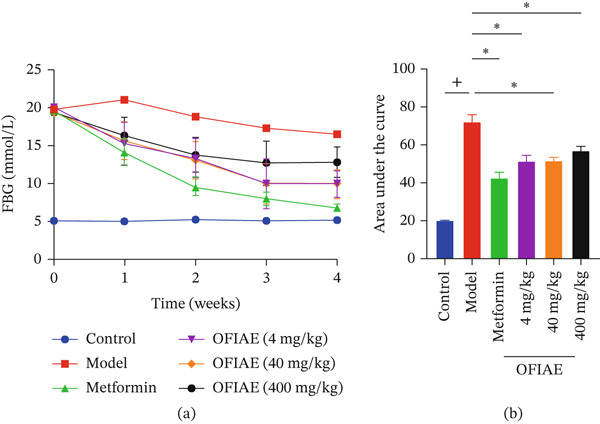
Effect of OFIAE on STZ‐induced diabetic rats. (a) Fasting blood glucose (FBG) levels of control and STZ‐induced diabetic rats. Displayed as mean ± SEM (*n* = 6). (b) Area under the curve of the various treatments. Rats were orally administered OFIAE (4, 40, or 400 mg/kg), metformin (300 mg/kg), or 5 mL/kg distilled water (control). A one‐way ANOVA with Dunnett′s post hoc test was used to compare groups. ^+^ *p* < 0.05 (control vs. model);  ^∗^ *p* < 0.05 (model vs. treatment groups). Each bar is the mean ± SEM, *N* = 6.

Figure 9a presents the time‐course changes in FBG levels of control and STZ‐induced diabetic rats over the 28‐day treatment period. Before treatment, diabetic model rats exhibited markedly elevated FBG levels (~18.5 ± 0.5 mmol/L) compared with the control group (*p* < 0.05) and showed persistently high FBG throughout the study period. Oral administration of OFIAE (4, 40, and 400 mg/kg) and metformin (300 mg/kg) resulted in a progressive reduction in FBG levels over time when compared with the diabetic model. The time‐course curve (Figure 9a) illustrates a gradual, sustained decline in FBG across the treatment duration, indicating improved glycemic control. At baseline, all treatment groups had FBG comparable to the model (*p* > 0.99), which significantly reduced between Week 1 and Week 3 in all OFIAE‐treated and metformin groups (*p* < 0.05). To quantitatively assess overall glycemic exposure during the study period, the area under the FBG–time curve (AUC) was calculated (Figure 9b). AUC analysis revealed a statistically significant reduction in cumulative FBG levels in OFIAE‐treated groups and the metformin group compared with the diabetic control (*p* < 0.05), confirming the glucose‐lowering effect of OFIAE. The progressive reduction in FBG levels demonstrates the antihyperglycemic potential of OFIAE and suggests improved long‐term glucose regulation in diabetic conditions.

### 3.7. Effects of OFIAE on Body Weight

**Figure 10:**
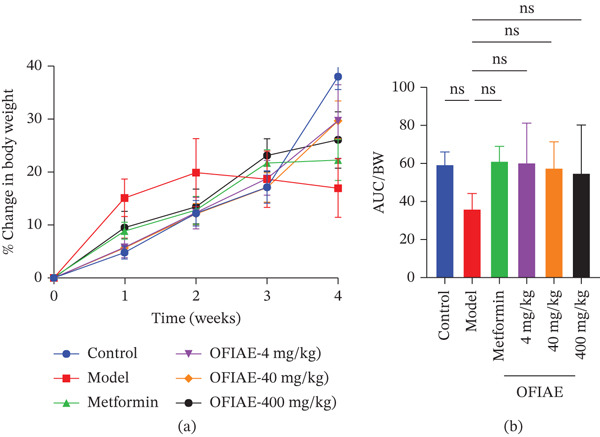
Effect of OFIAE on body weight and relative organ weight. (a) Percentage (%) change in body weight of control rats, STZ‐induced diabetic rats, and OFIAE‐treated rats. (b) Area under the curve for the various treatments. Rats were orally administered OFIAE (4, 40, or 400 mg/kg), metformin (300 mg/kg), or 5 mL/kg distilled water (control). A one‐way ANOVA with Dunnett′s post hoc test was used to compare groups. ^+^ *p* < 0.05 (control vs. model);  ^∗^ *p* < 0.05 (model vs. treatment groups). Each bar is the mean ± SEM, *N* = 6. ns = not significant.

Diabetic model rats exhibited a progressive decline in body weight over time, whereas rats treated with OFIAE (4, 40, and 400 mg/kg) and metformin (*p* < 0.05) showed attenuation of weight loss and gradual weight stabilization across the study duration. The time‐course curve (Figure 10a) illustrates longitudinal trends in body weight changes and is presented descriptively. To quantitatively evaluate overall changes in body weight throughout the treatment period, the area under the body weight–time curve (AUC) was calculated (Figure 10b). AUC analysis showed no statistically significant differences in cumulative body weight change between OFIAE‐treated groups and the diabetic control (*p* > 0.05).

### 3.8. HbA1c and Estimated Average Glucose (eAG)

**Figure 11:**
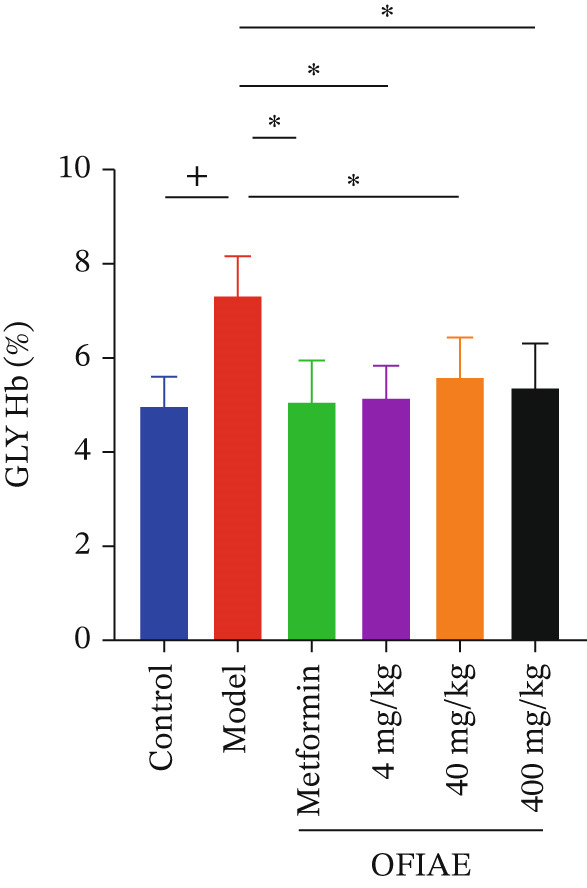
Effect of OFIAE on percentage glycated hemoglobin (HbA1c). Rats were orally administered OFIAE (4, 40, or 400 mg/kg), metformin (300 mg/kg), or 5 mL/kg distilled water (control). A one‐way ANOVA with Dunnett′s post hoc test was used to compare groups. ^+^ *p* < 0.05 (control vs. model);  ^∗^ *p* < 0.05 (model vs. treatment groups). Each bar is the mean ± SEM, *N* = 6.

The STZ‐induced diabetic model showed a substantial increase in glycation index (GH: 47.20 ± 5.41) and eAG (7.69 ± 0.79 mmol/L) compared to the control (GH: 30.69 ± 6.39, eAG: 5.29 ± 0.93 mmol/L), indicating poor glycemic control. Treatment with the reference markedly reduced these parameters (GH: 35.34 ± 12.77, eAG: 5.97 ± 1.86 mmol/L). OFIAE showed a reduction in HbA1c: OFIAE (4 mg/kg) (GH: 38.57 ± 10.28, eAG: 5.87 ± 1.23 mmol/L), OFIAE (40 mg/kg) (GH: 39.11 ± 10.34, 6.52 ± 1.51 mmol/L), and OFIAE (400 mg/kg) (GH: 40.28 ± 14.84, 6.69 ± 2.16 mmol/L), *p* < 0.05. Treatment with OFIAE (4, 40, and 400 mg/kg) resulted in a significant reduction in HbA1c levels compared with the diabetic model group. However, comparison among the three OFIAE doses revealed no clear graded or monotonic trend, indicating that the reduction in HbA1c was not dose‐dependent within the tested dose range. The observed effect was comparable across all OFIAE doses. The reduction in HbA1c levels indicates improved chronic glycemic control, reflecting sustained antihyperglycemic effects of OFIAE over the treatment period (Figure 11).

### 3.9. Histological Examination of the Pancreas

**Figure 12:**
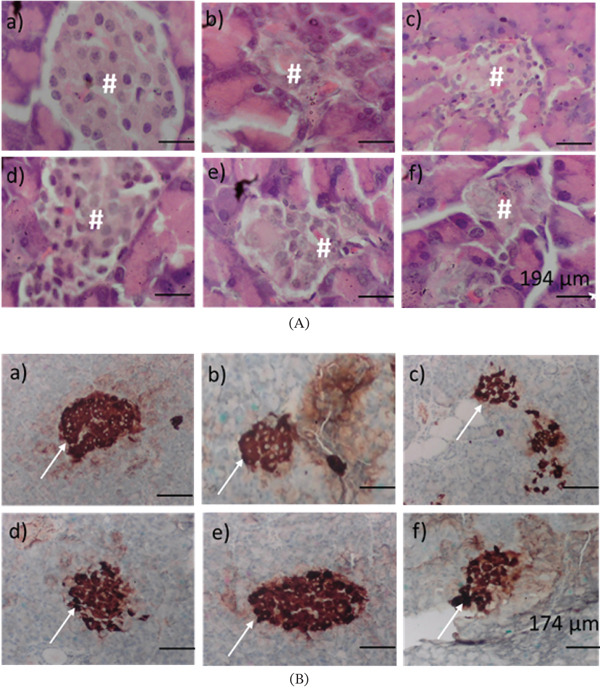
Effect of OFIAE on pancreatic histology and insulin secretion. (A) Photomicrographs of pancreatic tissue stained with hematoxylin and eosin (H&E) showing pancreatic islet cells (#), magnification ×400. (B) Immunohistochemical staining of insulin‐positive pancreatic beta cells (shown by arrows), magnification ×100. Images are representative of six pancreatic tissues in each group. (a) Control, (b) model, (c) metformin (300 mg/kg), (d) OFIAE (4 mg/kg), (e) OFIAE (40 mg/kg), and (f) OFIAE (400 mg/kg).

Compared to the control, the pancreatic sections of the model showed shrunken and necrotic islets of Langerhans. In contrast, OFIAE‐treated animals showed dose‐dependent restoration of islet histoarchitecture. The 40 mg/kg dose notably preserved islet integrity (Figure 12). Compared to the control, immunohistochemical analysis revealed reduced insulin‐positive staining in the model group (69.7%), based on percentage threshold values analyzed using ImageJ. Treatment with OFIAE improved *β*‐cell insulin expression, with the 4 and 40 mg/kg groups reaching 88.1% and 80.5% insulin‐positive areas, respectively, comparable to the control (88.2%). The 400 mg/kg group showed no additional benefit (78.5%), suggesting a plateau in *β*‐cell recovery (Figure 12).

### 3.10. Histological Examination of the Liver

**Figure 13:**
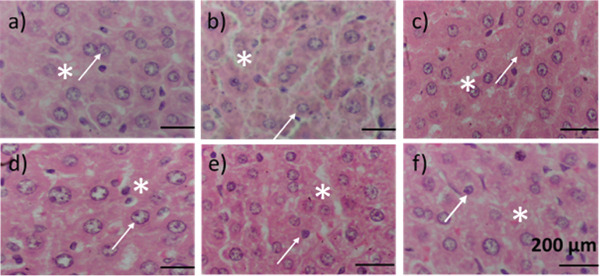
Effect of OFIAE on hepatic glucose storage. Representative liver tissues were stained with hematoxylin and eosin (H&E), magnification ×400. White arrows and asterisks represent hepatocytes and sinusoids, respectively. Images are representative of six hepatic tissues per group: (a) control, (b) model, (c) metformin (300 mg/kg), (d) OFIAE (4 mg/kg), (e) OFIAE (40 mg/kg), and (f) OFIAE (400 mg/kg).

The control group showed intact hepatic cords with radially arranged hepatocytes neighboring the central vein and well‐preserved sinusoidal spaces. In contrast, the diabetic model exhibited marked hepatocellular degeneration, disruption of the hepatic architecture, and sinusoidal dilation, hallmarks of diabetic hepatopathy. Treatment with the reference, metformin (300 mg/kg), showed moderate recovery of hepatic cords and reduced cellular degeneration. Rats treated with OFIAE (4 mg/kg) demonstrated mild improvement, while the OFIAE (40 mg/kg) group displayed near‐complete restoration of lobular architecture with hepatocytes arranged comparably to the normal control. However, the OFIAE (400 mg/kg) group showed some vacuolar changes and mild vascular congestion, suggesting a plateau or decline in protective effect at higher doses (Figure 13).

### 3.11. Liver Function Test

In STZ‐induced hyperglycemic rats, the diabetic model group demonstrated significantly elevated AST, ALT, ALP, GGT, and bilirubin levels (*p* < 0.05) compared to the normoglycemic control (Table 3). Treatment with OFIAE at all doses reduced these elevations, with the 40 mg/kg dose showing the most consistent normalization toward control values. Total protein fractions remained largely unchanged across groups.

**Table 3:** Effect of OFIAE on liver function of STZ‐induced diabetic rats.

Treatment group (*n* = 6)	Control	Model	Reference	OFIAE (4 mg/kg)	OFIAE (40 mg/kg)	OFIAE (400 mg/kg)
Total protein (g/L)	66.33 ± 2.367	70.17 ± 1.54	67.00 ± 1.528^∗^	70.00 ± 1.71	72.17 ± 1.25	71.17 ± 1.99
Albumin (g/L)	37.83 ± 1.80	38.17 ± 1.01	33.67 ± 1.23	36.50 ± 0.50	36.17 ± 0.40	37.00 ± 0.78
Globulin (g/L)	30.83 ± 1.35	34.33 ± 0.49	33.33 ± 1.43	33.33 ± 1.33	34.33 ± 1.28	30.00 ± 0.63^∗^
AST (U/L)	151.83 ± 2.96^+^	205.7 ± 8.51	151.7 ± 10.72^∗^	147.7 ± 10.42^∗^	120.5 ± 4.57∗	146.5 ± 7.77^∗^
ALT (U/L)	41.50 ± 2.67^+^	83.00 ± 3.39	82.83 ± 5.85	83.17 ± 4.18	61.33 ± 2.45^∗^	64.00 ± 3.84^∗^
GGT (U/L)	10.12 ± 0.55^+^	15.75 ± 1.11	6.33 ± 1.23^∗^	8.833 ± 1.01^∗^	9.17 ± 1.54^∗^	9.67 ± 1.15^∗^
ALP (U/L)	220.5 ± 7.48^+^	333.0 ± 18.33	432.5 ± 21.69^∗^	366.0 ± 30.33	242.3 ± 9.04^∗^	219.2 ± 9.48^∗^
Total bilirubin (*μ*mol/L)	3.02 ± 0.29^+^	4.80 ± 0.54	2.40 ± 0.23^∗^	2.85 ± 0.24^∗^	3.17 ± 0.40^∗^	3.45 ± 0.53
Direct bilirubin (*μ*mol/L)	2.97 ± 0.25^+^	4.47 ± 0.4394	1.82 ± 0.24^∗^	1.68 ± 0.20^∗^	2.67 ± 0.50^∗^	3.22 ± 0.51

*Note:* Total protein, albumin, globulin, AST, ALT, GGT, ALP, and total and direct bilirubin are indicators of liver function. Data is represented as mean ± SEM, *n* = 6. Significant differences between the model and treatment groups are designated by an asterisk (^+^ *p* < 0.05, control vs. model;  ^∗^ *p* < 0.05, model vs. treatment groups).

### 3.12. Semiquantitation of Liver Glycogen Storage

**Figure 14:**
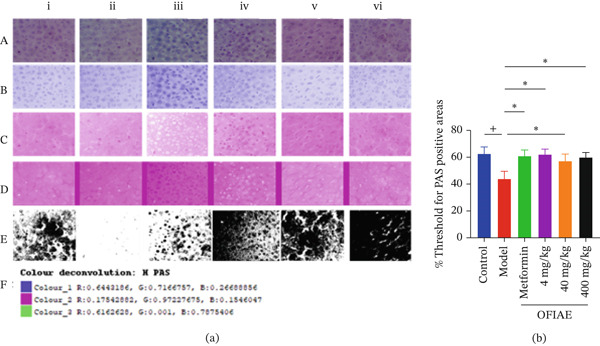
Semiquantitation of glycogen in PAS‐stained liver sections. (a) PAS‐stained liver sections showing glycogen distribution (magenta): (i) control, (ii) model, (iii) metformin (300 mg/kg), (iv) OFIAE (4 mg/kg), (v) OFIAE (40 mg/kg), and (vi) OFIAE (400 mg/kg). (A) Micrograph shows the original PAS‐stained liver, (B and C) processed images after color deconvolution using ImageJ, and (D and E) the inverted micrographs and glycogen distribution areas in black sections. (F) Vectors for color deconvolution. (b) Percentage threshold values of areas corresponding to glycogen‐positive areas. ^+^ *p* < 0.05 (control vs. model);  ^∗^ *p* < 0.05 (model vs. treatment groups). Images are the representatives of each group, *N* = 6. Each bar is the mean ± SEM, *N* = 6.

Hepatic glycogen accumulation in the control and STZ‐induced diabetic rats was evaluated using PAS staining. Liver sections from the diabetic model had markedly reduced PAS‐positive staining, indicating depleted glycogen reserves due to impaired insulin activity and glucose utilization. In contrast, treatment with OFIAE, particularly at 4 and 40 mg/kg, restored glycogen deposition as evidenced by intense magenta staining in hepatocytes. The results were comparable to those seen in the reference and control groups (Figure 14). Increased hepatic glycogen storage in treated groups indicates improved insulin action and enhanced glucose utilization, reflecting better metabolic control.

## 4. Discussion

The increasing global prevalence of DM, coupled with the high cost and side effects of current antidiabetic treatments, has intensified the search for safer and more cost‐effective therapeutic alternatives [36]. The current study investigated the antidiabetic properties of an aqueous extract of *OFI* cladode (OFIAE) and the possible mechanisms of action using in vitro and in vivo diabetic and/or nondiabetic models. OFIAE exhibited antihyperglycemic effects by increasing insulin secretion, promoting glycogen storage in the liver, and inhibiting digestive enzymes. These findings support its traditional use and highlight its potential as a complementary therapy for T2DM.

Hyperglycemia is a major hallmark of diabetes, and it underlies cachexia, weight loss, and both microvascular and macrovascular complications [37]. Therefore, its management is pivotal in regulating the diabetes pathophysiology. In this study, the blood glucose–lowering potential of OFIAE was assessed in normoglycemic rats using OGTT and in STZ–nicotinamide‐induced diabetic rats by monitoring the FBG weekly and assaying HbA1c at the endpoint. OFIAE demonstrated the potential to lower the postprandial rise of blood glucose in the normoglycemic rats after oral glucose load. The enhanced glucose tolerance observed during the OGTT suggests that OFIAE may improve postprandial glucose handling. OFIAE reduced blood glucose levels in the diabetic rats over 28 days and restored the euglycemic state, suggesting its antihyperglycemic effects. HbA1c is a stable, irreversible product formed when glucose in the bloodstream nonenzymatically binds to the N‐terminal valine of the *β*‐chain of hemoglobin A within red blood cells [38]. HbA1c reflects overall blood glucose over several weeks and is a reliable marker for assessing therapeutic efficacy [39]. In diabetes, the percentage of HbA1c rises (more than 6.5%). Treatment of the diabetic rats with OFIAE reduced HbA1c, suggestive of stabilization of glycemia and a potential delay in diabetes‐related complications. These results corroborate earlier reports on the hypoglycemic efficacy of *OFI* cladode and even fruit extracts [40, 41]. Additionally, weight loss, which is engendered by hyperglycemia, was also reversed by OFIAE in a dose‐dependent manner. Weight recovery promotes survival, reflecting improved energy metabolism, nutrient absorption, and overall health.

Blood glucose levels are tightly regulated through coordinated insulin secretion and peripheral glucose utilization, maintaining glucose homeostasis within a narrow range [42]. In healthy animals, pancreatic islets contain functional *β*‐cells that are responsible for insulin secretion. STZ‐induced diabetes is characterized by *β*‐cell damage and reduced insulin production, leading to hyperglycemia [43]. This study assessed the histoarchitecture of the islets of Langerhans (using H&E stain) as well as the insulin content of the islets (using immunostaining) in the STZ/NIC‐induced diabetic rats. Diabetic model rats in this study showed reduced islet size and disrupted islet architecture, consistent with *β*‐cell injury. OFIAE, notably at 40 mg/kg, preserved the histoarchitecture of the islets of Langerhans and also enhanced insulin secretion, as evidenced by increased insulin immunoreactivity in pancreatic islets of OFIAE‐treated diabetic rats. These observations highlight the potential of OFIAE to recover the pancreatic *β*‐cells, which are often damaged in both clinical and experimental diabetes, and confirm OFIAE′s insulinotropic effects. However, serum insulin level was not directly quantified, and therefore, these findings should be interpreted as indirect evidence of improved pancreatic function.

Also, under normal conditions, excess circulating glucose is stored in the liver in the form of glycogen through insulin‐mediated processes [44]. This balance is crucial for maintaining normal blood glucose levels. In DM, impaired insulin action reduces glycogen synthesis and promotes glycogen depletion in hepatic tissue. Glycogen in the hepatocytes can be stained using PAS, and the staining intensity can be semiquantified to reflect the glycogen storage potential of the hepatocytes [45]. PAS staining of liver sections in the present study showed reduced glycogen deposition in diabetic model rats, indicating impaired hepatic glucose storage. Treatment with OFIAE increased hepatic glycogen accumulation, particularly at 40 mg/kg. The increased glycogen deposition suggests improved hepatic glucose utilization and storage, which is consistent with improved metabolic regulation. Preservation of liver hepatocytes and liver biochemical parameters by OFIAE also supports the observed enhanced liver function.

One of the prerequisites of glycogen storage is glucose uptake, a process that is mediated by glucose transporters [46]. This study also assessed the potential of OFIAE to promote glucose uptake using yeast cells, a model that lacks insulin receptors but expresses glucose transporters analogous to mammalian GLUTs, especially GLUT2 and GLUT4. The increased glucose uptake observed in yeast cells suggests that OFIAE contains compounds capable of facilitating cellular glucose transport, possibly via insulin‐independent pathways, suggestive that OFIAE may utilize insulin‐independent‐mediated GLUT‐mediated glucose transport, peripheral glucose uptake, and utilization [47]. These results suggest that OFIAE may promote glucose uptake through both insulin‐dependent and insulin‐independent mechanisms.

Additionally, carbohydrate digestion by *α*‐amylase and *α*‐glucosidase plays an important role in postprandial glucose regulation. Inhibition of these enzymes delays the breakdown of complex carbohydrates to glucose and thus reduces the postprandial glucose spike [48]. OFIAE demonstrated moderate inhibition of *α*‐amylase and *α*‐glucosidase in vitro. This observation suggests that delayed digestion of complex carbohydrates may contribute to the antihyperglycemic effects observed in vivo, although enzyme inhibition was not directly evaluated under physiological conditions.

The antihyperglycemic potential of OFIAE may be attributed to the presence of bioactive secondary metabolites in OFIAE. Preliminary phytochemical screening revealed flavonoids, alkaloids, and phenolic compounds, all of which have been widely reported to exert antidiabetic effects through multiple mechanisms. Flavonoids, for instance, have been shown to enhance insulin secretion, improve insulin sensitivity, and stimulate glucose uptake in peripheral tissues [49, 50]. Alkaloids may contribute by inhibiting intestinal glucose absorption and modulating enzymes involved in carbohydrate metabolism, such as *α*‐glucosidase and *α*‐amylase [51, 52]. Phenolic compounds are known to possess strong antioxidant properties, which help to reduce oxidative stress, a major contributor to *β*‐cell dysfunction and insulin resistance in diabetes [53]. Together, these compounds may act synergistically to regulate blood glucose levels and preserve pancreatic and hepatic function.

A notable finding of this study was that the 4 and 40 mg/kg doses consistently produced greater antihyperglycemic effects than the 400 mg/kg dose across multiple parameters, including fasting glucose, HbA1c, hepatic glycogen deposition, and pancreatic morphology. Several pharmacological explanations may account for the reduced efficacy observed at the highest dose. One possibility is phytochemical antagonism, whereby certain constituents exert opposing metabolic effects when present at higher concentrations, thereby reducing overall activity [54]. Another explanation may involve hormesis, a phenomenon in which low or moderate doses produce beneficial biological responses while higher doses lead to diminished effects [55]. Additionally, *OFI* cladodes contain substantial amounts of soluble dietary fiber and mucilage, which increase viscosity in the gastrointestinal tract [56]. At higher doses, increased viscosity may impair intestinal absorption of bioactive compounds, thereby reducing systemic pharmacological activity [57]. High extract concentrations may also influence palatability or gastrointestinal function, potentially affecting nutrient utilization and metabolic outcomes. From a pharmacodynamic perspective, the nonlinear dose–response relationship suggests that increasing the dose of OFIAE beyond the optimal range does not enhance therapeutic benefit and may reduce efficacy. This finding is important for the rational development of plant‐based antidiabetic agents, as it highlights the need for dose optimization rather than simple dose escalation.

Although the present findings demonstrate antihyperglycemic activity in an experimental model, translation to clinical use requires careful consideration. Using body surface area conversion, the effective dose of 40 mg/kg in rats corresponds approximately to a human‐equivalent dose of about 6–7 mg/kg (approximately 400 mg/day for a 70‐kg adult). This estimate suggests that pharmacologically relevant doses may be achievable in humans; however, controlled clinical studies are required to establish safety and efficacy in patients with diabetes.

Overall, the present findings suggest that OFIAE possesses significant antihyperglycemic activity in experimental diabetes. The results are consistent with improvements in glucose handling, pancreatic integrity, and hepatic glycogen storage, although the precise molecular mechanisms remain to be clarified. Further studies incorporating phytochemical standardization, mechanistic investigations, and clinical evaluation are required to define the therapeutic potential of OFIAE more clearly.

## 5. Conclusion

*OFI* cladode extract (OFIAE) demonstrated antihyperglycemic effects possibly through inhibition of carbohydrases (*α*‐amylase and *α*‐glucosidase), enhancing peripheral glucose uptake, increasing insulin secretion, and promoting hepatic glycogen storage. These findings support the traditional use of *OFI* in diabetes management and indicate that the OFIAE possesses biologically relevant glucose‐lowering activity. Also, OFIAE demonstrated dose‐independent antihyperglycemic effect with the middle dose (40 mg/kg) producing a comparatively enhanced antihyperglycemic effect relative to the high dose (400 mg/kg). This finding highlights the need for establishing a safe human dose (SHD) for OFIAE as part of dose optimization in the development of plant‐based antidiabetic therapies such as OFIAE.

## 6. Limitations

Despite the promising findings, the study could still benefit from the following. First, the proposed mechanisms underlying the antihyperglycemic effects were inferred from indirect evidence, as molecular pathways related to insulin signaling, glucose transport, and carbohydrate metabolism were not directly investigated. Second, OFIAE was evaluated as a crude aqueous preparation, and the specific bioactive compounds responsible for the observed effects were not isolated and quantified. Additionally, the pharmacokinetic profile of OFIAE and chronic toxicity assessment on OFIAE were not assessed. Although the STZ–nicotinamide‐induced diabetic rat model provides a valuable experimental basis for testing efficacy and mechanism of action of antidiabetic agents such as OFIAE, there is still a need for the use of more refined and specific diabetes models (use of transgenic diabetic rats, commercially available pancreatic beta cells, etc.) to confirm OFIAE′s antidiabetic properties. Therefore, future studies on OFIAE should prioritize the standardization of phytocompounds in OFIAE, the establishment of SHD, and controlled clinical trials to better establish the therapeutic potential of OFIAE in humans.

**Nomenclature**
ALP: alkaline phosphatase
ALT: alanine aminotransferase
AST: aspartate aminotransferase
FBG: fasting blood glucose
NIC: nicotinamide
OFIAE: *Opuntia ficus-indica* aqueous extract
OGTT: oral glucose tolerance test
STZ: streptozotocin

## Author Contributions

N.E.M. performed in vitro and in vivo experiments, collected data, and participated in data analysis; E.A.A. analyzed data and performed supervision; O.M. performed in vivo experiments; J.K.A. performed phytochemical analysis; S.K. supervised experiments and reviewed the final draft; A.B. conceptualized, supervised, and reviewed the final draft for intellectual content.

## Funding

This work was supported by Partnering for Health Professional Training in African Universities (P4PHT).

## Conflicts of Interest

The authors declare no conflicts of interest.

## Data Availability

The data that support the findings of this study are available from the corresponding author upon reasonable request.
